# Photo-rechargeable Li-Ion Batteries with Lead-Free Double-Perovskite Halide Cs_2_NaBiI_6_

**DOI:** 10.1021/acsami.5c06043

**Published:** 2025-07-28

**Authors:** Neha Tewari, Davy Lam, Pui Kei Ko, Pai Geng, Herman H. Y. Sung, C.-H. Angus Li, Ian Duncan Williams, Jonathan E. Halpert

**Affiliations:** Department of Chemistry, 58207 Hong Kong University of Science and Technology, Clear Water Bay Road, Kowloon, Hong Kong SAR, 999077

**Keywords:** photobattery, photoelectrode, Li-ion, PHBAT, solar battery, double perovskite, Cs_2_NaBiI_6_

## Abstract

Perovskite halides are promising materials for bifunctional devices that can achieve both photovoltaic energy generation and energy storage. Here, a lead-free all-inorganic double-perovskite halide, 3D Cs_2_NaBiI_6_, has been investigated as both electrode and photoelectrode active layer in a Li-ion battery. This nontoxic, environmentally friendly material shows impressive lithium storage capacity performance, as compared to previous perovskite photoelectrodes, such as Cs_3_Bi_2_I_9._ Li-ion batteries using this material, in a standard coin cell configuration, achieved a champion first charge specific capacity of 450 mAhg^–1^ and 150 mAhg^–1^ after 90 cycles, with devices functioning well beyond 500 cycles. Furthermore, bifunctional performance with energy harvesting and energy storage properties was achieved by fabricating photobatteries (PHBATs) in a modified coin cell. PHBATs using Cs_2_NaBiI_6_ represent the first double perovskite to be used in this application and achieved a “first charge” light conversion efficiency (LCE) of 0.27%, higher than most photoelectrode materials reported to date and with better stability than previously studied bismuth perovskites.

## Graphical abstract

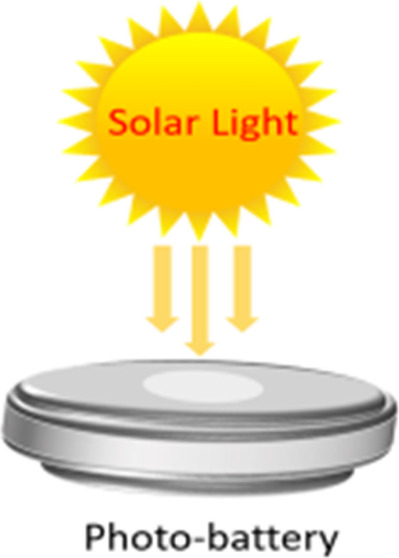

## 1 Introduction

The current demand for energy products has reignited interest in materials that can combine energy harvesting and storage in a single integrated device.^1–3^ One way to achieve such a device is by integrating light absorbing photovoltaic materials into a battery to create a photobattery (PHBAT).^4,5^ While existing solar cells and batteries can be wired together, a single bifunctional device alone could have significant advantages in energy density, by weight and volume, if it can be made to be efficient.^6^ Although photobatteries were first reported in 1976,^7^ materials that can serve as both the solar material and charge storage layer are rare and the power conversion efficiency was generally low.^8,9^ Very recently, advances in the literature suggest a promising new start for this technology,^10–13^ including, for example, reports of “first charge” LCEs in PHBATs approaching 1%.^6,14^

Metal halide perovskites have also been explored for photobatteries,^2,8^ since they have a high absorption coefficient, exhibit free charge generation, and have a defect tolerance that enables stable ion doping into the crystal lattice.^15,16^ Lead-free perovskites have been demonstrated, including our own previously demonstrated PHBAT using hexagonal Cs_3_Bi_2_I_9_ nanocrystals.^17^ However, the poor stability of the active layers in perovskites is still a limitation, and new perovskites are needed that show superior lithium-ion storage along with good performance, efficiency, and environmental compatibility. Double perovskites offer an alternative class of materials with a large number of possible structures. The double-perovskite structure is derived by replacing Pb^2+^ in an ABX_3_ structure with alternating monovalent and trivalent cations.^18^ A wide range of lead-free double perovskites with varying optical and electronic properties have been synthesized with an A_2_B^I^B^III^X_6_ structure where A = Cs^+^, Rb^+^; B^I^ = Na^+^, K^+^, Li^+^, Ag^+^, Cu+; B^III^ = In^3+^, Bi^3+^, Sb^3+^.^19,20^ Double-perovskite halide solar cells have also been reported;^21,22^ however they can also be used as the Li^+^ storage layer electrodes in Li-ion batteries (LIBs).^23,24^ Only a few examples of double-perovskite LIBs exist, including Cs_2_NaBiCl_6_, which was reported as an anode material capable of achieving a stable specific capacity of approximately 300 mAhg^–1^ with over 99% Coulombic efficiency,^24^ and Cs_2_AgBiBr_6_ double perovskite, which was used to store solar energy in a photoelectrochemical system incorporating cobalt complexes and methyl viologen redox mediators.^25^

Here we present the first use of a double perovskite in a lithium-ion PHBAT by using Cs_2_NaBiI_6_ as the active layer. This material was chosen due to its low band gap, high resistance to moisture and oxygen in ambient air, and ability to insert Li^+^ into the crystal structure without degradation. The standard Li-ion batteries showed a champion first charge specific capacity of 450 mAhg^–1^, 150 mAhg^–1^ after 90 cycles, and <100 mAhg^–1^ after 500 cycles. Furthermore, bifunctional performance, including both energy harvesting and energy storage, was demonstrated in these photobatteries (PHBATs) and a “first-charge” light conversion efficiency (LCE) of 0.27% was achieved. This is higher than the LCE of most reported photoelectrodes such as V_2_O_5_ photocathodes, achieving 0.22% for LIBs^14^ and 0.2% for MoS_2_/ZnO photocathodes in zinc ion batteries (ZIBs) under 1 sun.^26^

## 2 Results and Discussion

The double-perovskite halide Cs_2_NaBiI_6_ was synthesized using a one-step hydrothermal method (see Methods section for complete details). As illustrated in Figure 1a, the crystal structure of Cs_2_NaBiI_6_ is cubic, exhibiting a typical 3D perovskite structure with corner-shared NaI_6_ and BiI_6_ octahedra in all three directions. Field-emission scanning electron microscopy (FESEM) images in Figure 1b show smooth, faceted 3D Cs_2_NaBiI_6_ microcrystals of a few micrometers in width and length. The powder-XRD patterns are compared to those previously reported for Cs_2_NaBiI_6_ powder^27^ in Figure 1c. These show similar peaks to the comparable double perovskite, (MA)_2_AgBiI_6_, where four peaks are observed at 13.8°, 25.2°, 27.6°, and 32.3° and match well to those reported for Cs_2_NaBiI_6._ ^28^ This is consistent with the stated results, and our XRD patterns indicate the development of double perovskites with hexagonal crystals and a symmetric space group of *P*6_3_/*mmc*. The absence of starting materials in the diffraction patterns suggests a high level of product and phase purity. The strong optical absorption and very broad excitonic absorption peak at 500 nm give an estimated direct band gap of 2.12 eV and indirect band gap of 1.42 eV, consistent with literature values^27–29^ and as shown in Figure 1d. The band gap values make this material a suitable choice for harvesting UV and visible wavelengths of nearly 600 nm. A schematic representation of the PHBAT assembly using Cs_2_NaBiI_6_ as the active layer is shown in Figure S1.

**Figure 1:**
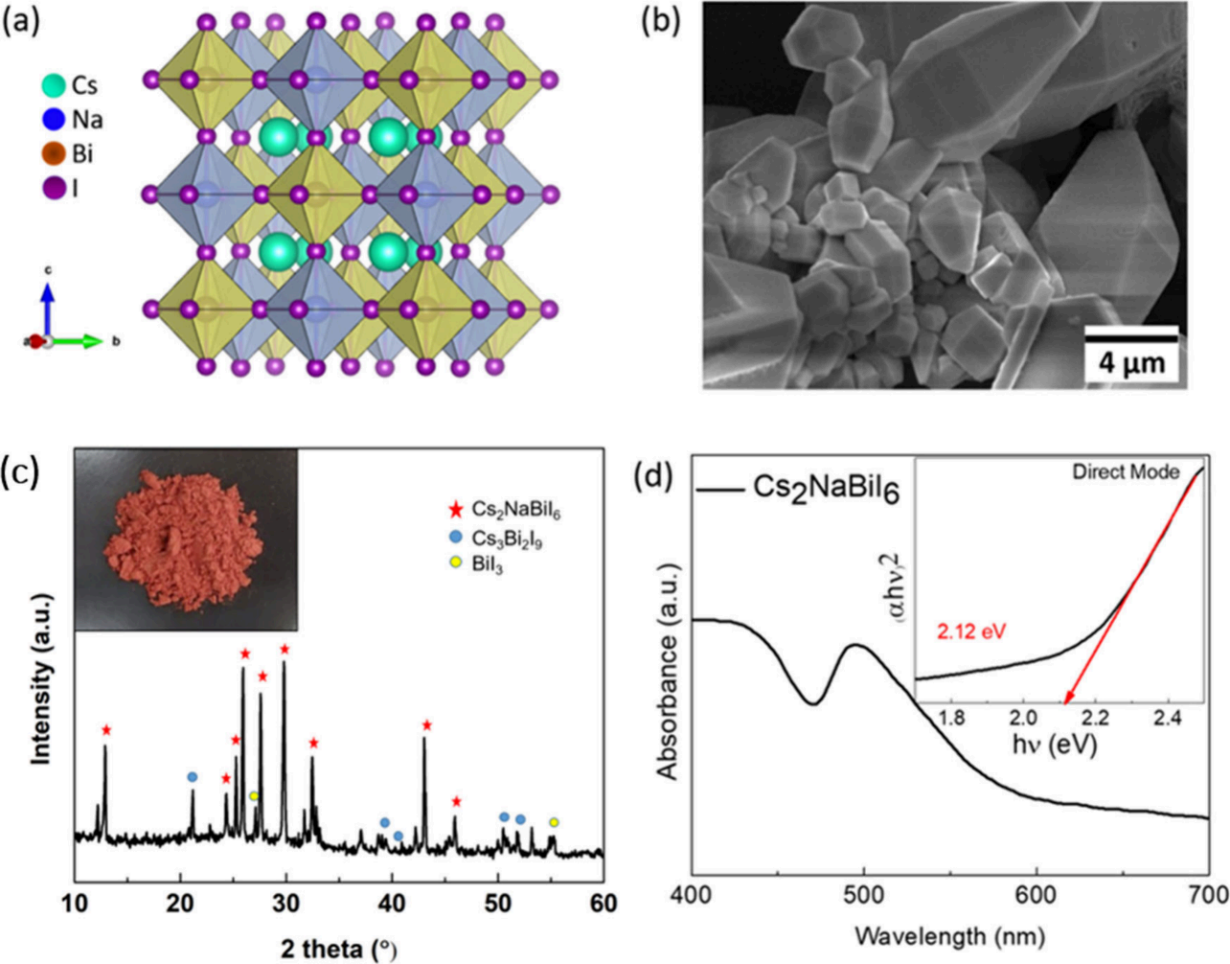
(a) Crystal structure of Cs_2_NaBiI_6_, (b) SEM image of 3D Cs_2_NaBiI_6_ multiple micron-sized shuttle-like crystals, and (c) XRD pattern of the as-prepared Cs_2_NaBiI_6_ powder, showing impurities BiI_3_ and Cs_3_Bi_2_I_9_. Red dots denote characteristic peaks of Cs_2_NaBiI_6_. (d) UV–vis spectrum of the Cs_2_NaBiI_6_ thin film. Inset shows the Tauc plot with an optical band edge at 2.12 eV.

Standard (non-PHBAT) Li-ion coin cells were fabricated using Cs_2_NaBiI_6_, and Li^+^ storage was measured via electrochemical analysis (see Methods). Coin cells of type CR2450 were prepared using established methods^6^ in a half-cell configuration with Li foil as the counter and reference electrode. The galvanostatic charge–discharge (GCD) curves and cyclic voltammetry in the range of 2.50–0.01 V vs Li/Li^+^ are shown in Figure 2a and 2b, respectively. These were measured with an initial current density of 50 mAg^–1^ for the first five cycles and 100 mAg^–1^ for all subsequent cycles. The initial specific discharge capacity was observed to be 460 mAhg^–1^, following which an irreversible loss of capacity was observed due to the creation of the solid electrolyte interface and the conversion of Bi^3+^ to Bi^0^ in the perovskite structure.^17^ Irreversible Bi loss and SEI formation were also reported in previously published reports^17,30^ and for bismuth electrodes used in Li-ion (nonphoto-) batteries.^31^ Although capacity values after the first cycle declined marginally over time, the material displayed an impressive specific capacity of 150 mAhg^–1^ even after 90 cycles (Figure 2c). Figure S2 illustrates the system’s performance across 500 cycles, indicating remarkable long-term cycling stability.

**Figure 2:**
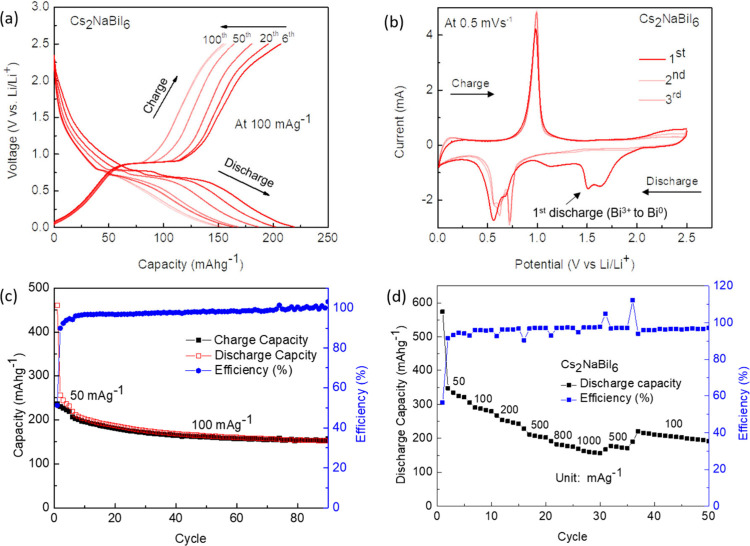
Electrochemical performance of Cs_2_NaBiI_6_ electrodes in lithium-ion batteries with a copper current collector. (a) Galvanostatic charge–discharge curves in the voltage range of 2.5–0.01 V vs Li/Li^+^ at 100 mAg^–1^ (cycle numbers and directions labeled) with the first 5 cycles at 50 mAg^–1^. (b) Cyclic voltammetry scans of Cs_2_NaBiI_6_ vs Li for the first 3 cycles at a voltage range of 2.5–0.01 V vs Li/Li^+^ at a scan rate of 0.5 mVs^–1^ (cycles and scan directions indicated). (c) Long-term cycle stability vs Li/Li^+^ at 100 mAg^–1^ with the first 5 cycles at 50 mAg^–1^. (d) Rate performance at varying current densities. Measurement error on cycle stability and rate performance measurements (capacity, efficiency) were less than ±0.1% of value.

Cyclic voltammetry was also used to investigate the lithiation and delithiation mechanisms in a potential window of 2.50–0.01 V vs Li/Li^+^ at a scan rate of 0.50 mVs^–1^. In Figure 2b, there is an irreversible broad peak between 1.0 and 2.0 V versus Li/Li^+^ in the first cycle of cathodic scanning that vanishes in subsequent cycles. This is due to the creation of a solid electrolyte interphase (SEI) layer on the surface of the perovskite halide and the reduction of Bi^3+^ in Cs_2_NaBiI_6_ to Bi^0^, which causes an irreversible loss of capacity during the first cycle. The reversible cathodic scan in the CV reveals two significant peaks. The low potential peaks, which range from 0.7 to 0.5 V, indicate that LiBi and Li_3_Bi, respectively, were formed, as was observed in previous studies of Bi Li-ion batteries.^30^ The steep oxidation peak seen at 0.9 V is due to the dealloying of Li_3_Bi into Bi^0^ and Li^+^. There is a strong correlation between the lithiation voltage obtained from the charge–discharge plots and the cyclic voltammetry plots. In the rate performance plot, in Figure 2d, Cs_2_NaBiI_6_ was subjected to varied current densities of 50 mAg^–1^, 100 mAg^–1^, 200 mAg^–1^, 500 mAg^–1^, 800 mAg^–1^, and 1000 mAg^–1^. Structural analysis was undertaken to determine whether the original material is reformed via cycling or if any structural changes take place.

Ex situ X-ray diffraction (XRD) has been shown to be effective in analyzing the lithiation process in a variety of electrode materials and was conducted to investigate the mechanism of lithium storage. The cells were discharged to various potential points in the first discharge cycle at 100 mAg^–1^ before being disassembled for analysis. After dissembling the cells, the electrodes were rinsed with DMC (dimethyl carbonate) to wash off the salt deposition on the electrode surface, since unwashed electrodes could have leftover crystallized LiPF_6_ or nonvolatile solvents that are challenging to separate from the SEI or intercalated Li components.^32^ These were dried at 60 °C before analysis. Ex situ XRD studies conducted at specific potentials during the first discharge cycle are shown in Figure S3a. As illustrated in Figure S3b, when the potential dropped from 2.5 V (pristine) to 1.6 V, a new peak at 27.26° arises, which is linked to the primary (012) peak of Bi (JCPDS Card No. 85-1329). This peak signifies the presence of metallic Bi and confirms the conversion reaction that leads to the conversion of Bi^3+^ in the perovskite to Bi^0^. Further discharge from 1.6 to 0.64 V shows that a LiBi phase appears and the intensity of the peak further increases from 0.64 V to the fully discharged state (∼0.01 V vs Li/Li^+^). This suggests that Bi^0^ combines with Li-ion to generate Li–Bi with a portion of the LiBi phase transforming into a Li_3_Bi phase as seen in eq 3.

The lithiation process in Cs_2_NaBiI_6_ adheres to a distinctly defined multistep sequence as shown in eqs 1–3. Initially, lithium ions intercalate into the double-perovskite structure, yielding Li_*x*_Cs_2_NaBiI_6_ (eq 1). This is followed by a conversion process, in which Bi^3+^ is reduced to metallic Bi^0^, disassembling the original perovskite structure. The resultant metallic bismuth gradually alloys with lithium to form LiBi (eq 2) and Li_3_Bi phases (eq 3), indicated by the characteristic CV peaks at 0.7 and 0.5 V, respectively. This mechanism clarifies the high initial discharge capacity (∼450 mAh/g) and the subsequent capacity stabilization linked to Li–Bi alloying reactions, with the irreversible capacity loss in the first cycle attributed to the conversion reaction and the formation of the solid electrolyte interphase (SEI). Several Bi-based materials, including bismuth oxyhalide, Bi_2_Se_3_, and BiVO_4_, undergo Li-ion storage via a conversion and alloying mechanism showing similar XRD peaks.^30,33,34^

During discharge:

Lithiation:

1 $$\mathrm{Li}+{\mathrm{Cs}}_{2}{\mathrm{NaBiI}}_{6}\to {\mathrm{Li}}_{x}{\mathrm{Cs}}_{2}{\mathrm{NaBiI}}_{6}$$

The process of lithiation is followed by conversion of perovskite Bi^3+^ to Bi^0^.

Alloying:

2 $$Bi+Li^{+}+e^{-}\to LiBi$$

3 $$LiBi+2Li^{+}+2e^{-}\to Li_{3}Bi$$

Following the successful fabrication and analysis of standard Li-ion batteries employing the double-perovskite electrode, we analyzed Cs_2_NaBiI_6_ as a photoelectrode in a PHBAT using a coin cell with a light window and other practical modifications. Copper, the current collector used as an opaque current collector in the Li-ion battery, cannot be used in a photoelectrode, as it would not let light pass through to the absorbing layer. Hence, we switched from copper (Cu) to carbon fiber (CF) as a porous and transparent current collector. The electrode was fabricated by combining Cs_2_NaBiI_6_, PCBM, and PVDF in a 70:20:10 weight ratio in NMP (1-methyl-2-pyrrolidinone) and then drop-casting 50 μL of the slurry onto the CF layer (see Supporting Information for device details). Figure S4b shows the SEM image of the electrode deposited on CF showing the hexagonal perovskite plate-like structures attached to the fibrous nonwoven carbon paper. Before the photoelectrochemical measurements were done, we analyzed the performance of the Cs_2_NaBiI_6_ photoelectrode with CF as a standard (nonphoto) battery. For that, measurements including galvanostatic charge–discharge (GCD) and cyclic voltammetry (CV) were performed, with results shown in Figure S5. The voltage window was narrowed down from 2.50–0.01 V vs Li/Li^+^ to 2.50–0.20 V vs Li/Li^+^. This was done because CF is carbon based and hence reacts with Li at low voltage, but charges stored at these potentials carry very little energy and contribute little to the energy storage efficiency. CF does contribute a small amount of storage above 0.20 V; however, this was negligible compared with that obtained for CNBI on CF electrodes. Figure S6 shows the galvanostatic charge–discharge curves for only CF vs Li for 250 cycles with discharge capacity values lower than 25 mAhg^–1^. The GCD curves in Figure S5a were conducted at 200 mAg^–1^ with the first 10 cycles at 100 mAg^–1^. The initial discharge cycle shows an irreversible value of 540 mAhg^–1^, which, as explained above, is due to the conversion reaction (which leads to the formation of metallic Bi) and the formation of a solid-electrolyte interphase (SEI). The charge–discharge cycle shows a stable performance with discharge and charge capacities of 270 mAhg^–1^ even after 80 cycles. When subjected to varying current densities of 50 mAg^–1^, 100 mAg^–1^, 200 mAg^–1^, 500 mAg^–1^, 800 mAg^–1^, and 1000 mAg^–1^, an impressive rate performance was achieved. Figure S5d shows retention approaching 100% in specific capacity (319 mAg^–1^) at 100 mAg^–1^ after cycling even at higher current densities up to 1000 mAg^–1^.

Apart from the performance of the (nonphoto-) battery with CF as the current collector, we tested the photoactivity of the Cs_2_NaBiI_6_ electrode. Chronoamperometry was conducted in dark and light circumstances against Li metal to demonstrate the effect of light on the value of current generated. As expected, the current increases when illuminated (by a xenon lamp at ∼1 sun) and the value of the current immediately falls when kept in the dark. Figure 3a depicts an increase in current responsiveness under illumination at an applied voltage of 0 V, applying alternate intervals of 8 min of light and 8 min of darkness. This demonstrates that mechanisms inside the Cs_2_NaBiI_6_ PHBAT are promoting the transport of photoinduced electrons. Additionally, as a demonstration, a 1.5 V digital stopwatch was powered with a PHBAT that is exclusively charged by light, as shown in Figure 3b.

**Figure 3:**
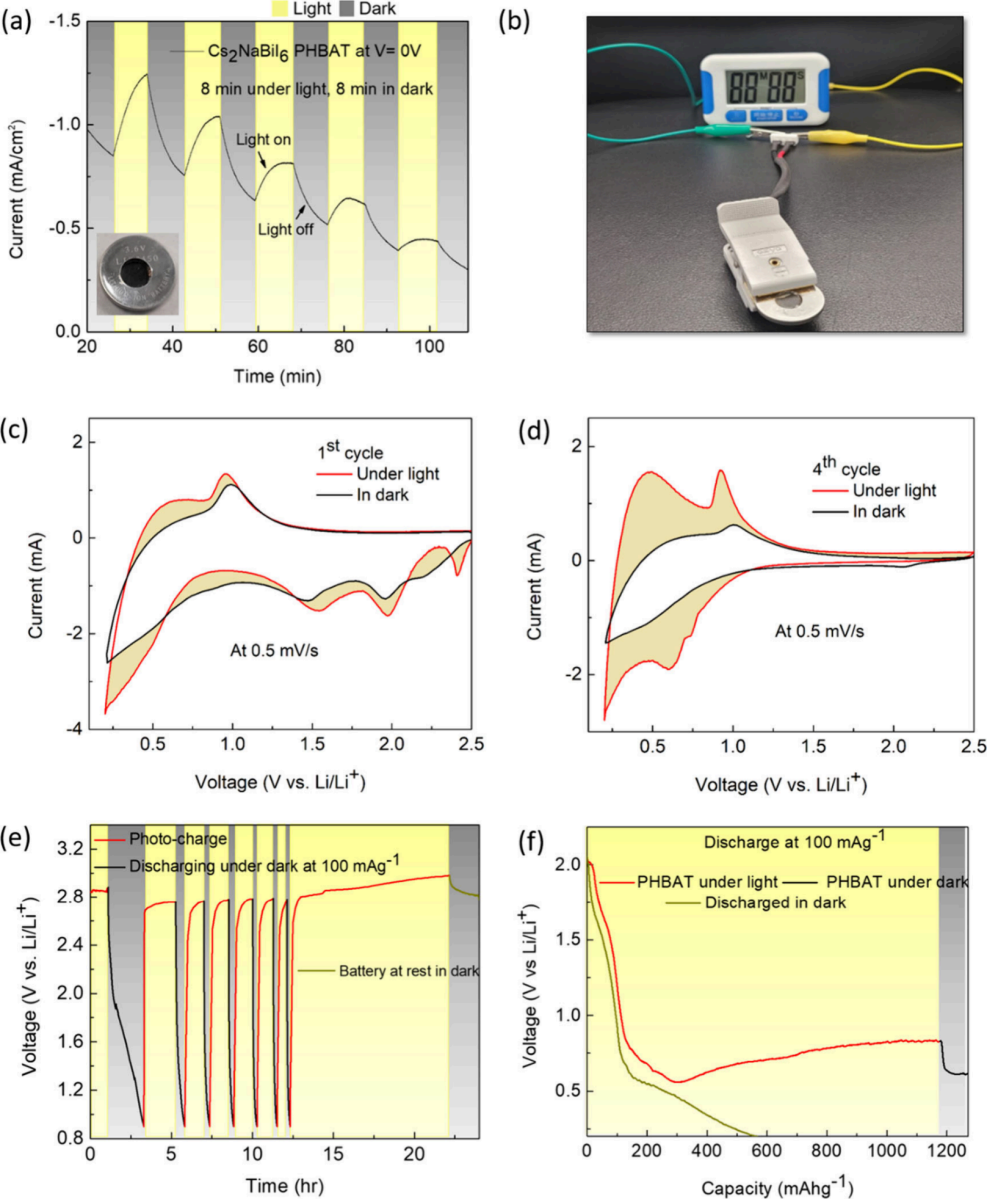
Photoelectrochemical analysis of Cs_2_NaBiI_6_. (a) Chronoamperometry response of the Cs_2_NaBiI_6_ photoelectrode with CF current collector and Li counter electrode at 0 V applied bias with alternating 8 min light (1 sun) and 8 min dark intervals. (b) A 1.5 V digital stopwatch powered with the PHBAT that is exclusively charged by light. CV curves at 0.5 mV s^–1^ in the dark and illuminated (under 1 sun) conditions over a range of 0.2–2.5 V vs Li/Li^+^; CV curves for the (c) 1st and (d) 4th cycle. (e) Photocharging of PHBAT under white light illumination at 1 sun intensity and discharge at a current density of 100 mAg^–1^. The regions highlighted in yellow denote periods of photocharging under illumination of 1 sun. The red lines indicate photocharging phases, the black lines denote galvanostatic discharge in darkness, and the green lines reflect battery rest in the dark. (f) Discharge curves of CF-PHBATs under light and dark conditions at a current density of 100 mAg^–1^ showing that the capacity can be arbitrarily determined by the “light on” time when the battery is discharged under conditions where the white light intensity and conversion efficiency are sufficient to compensate for the current lost during discharge.

The electrochemical responses of photo-LIBs are analyzed under dark and light conditions using cyclic voltammetry (CV), as shown in Figure 3c. The initial cycle shows a notable irreversible cathodic peak above 1.0 V versus Li/Li^+^, suggesting the conversion reaction from Bi^3+^ to Bi^0^ and the formation of the solid electrolyte interphase (SEI). The reduced visibility of Li–Bi alloying processes is due to the employment of CF as the current collector instead of Cu, as CF reacts with Li at lower voltages. The voltage range was limited to 2.5–0.2 V vs Li/Li^+^ to minimize CF interference, while allowing for the observation of critical electrochemical processes. The photosensitivity of the electrodes markedly increases the peak currents during both cathodic and anodic sweeps when exposed to white light, which is due to the photocharging effect and the photogeneration of charge carriers. In the fourth cycle (Figure 3d), the irreversible conversion peak above 1.0 V has completely disappeared, and the CV profile clearly shows the reversible Li–Bi alloying and dealloying peaks. The alloying peaks at 0.5–0.7 V and the dealloying peak around 0.9 V are clearly defined and show significant amplification under illumination, indicating improved reaction kinetics and charge transfer when exposed to light. The CV profile is consistently displayed for the fourth cycle in both light and darkness, suggesting that this effect is a bulk phenomenon rather than a surface one.

Following that, the PHBAT’s light charging capabilities are evaluated without using any external current. The open circuit voltage (OCV) of the battery was measured while it was photocharged briefly at 1 sun and then galvanostatically discharged in the dark at 100 mAg^–1^. Figure 3e demonstrates the PHBAT performance. Here, the discharge is limited to 0.9 V in order to avoid Li–Bi alloy formation in the perovskite, which can further lead to degradation of the electrode. The first discharge cycle to 0.9 V at 100 mAg^–1^ was followed by photocharge (without an external load), and this was performed for 7 cycles. The capacity drops somewhat after the first cycle, however photocharging is very clearly observed after each cycle as the voltage drop was minimal. After the seventh discharge, we kept the battery under a light intensity of 1 sun for a longer duration. We observed that the voltage shows constant gradual increases and rises above 2.9 V when kept under light. As soon as we turn off the light, a sudden voltage drop is observed (curve shown in light blue), proving that the increased voltage was due to the device capturing photogenerated charge carriers.

This experiment can also be used to calculate the light-conversion efficiency (LCE), which was 0.27% for the “first charge” efficiency of the champion device under the illumination of 1 sun (Figure S7 and Table S1). This value is greater than previously published photo-rechargeable perovskite LIBs, which have generally ranged from 0.03% to 0.22% under 1 sun excitation,^8,9,14,35^ though slightly lower than the champion LCE of our previously reported Cs_3_Bi_2_I_9_ PHBAT.^17^ In contrast, however, the Cs_2_NaBiI_6_ PHBATs in this work attain higher capacity, impressive rate capability, and greatly increased cycling stability (the discharge capacity achieved with the (nonphoto) Cs_2_NaBiI_6_ battery was 152 mAhg^–1^ as compared to 47 mAhg^–1^ achieved with Cs_3_Bi_2_I_9_ after 90 cycles).^17^ Two PHBATs, one discharged under light and the other in the dark, are shown in Figure 3f. We can observe the increased capacity in the PHBAT discharging under illumination as compared to the PHBAT discharging in the dark. This is because the PHBAT discharging under light undergoes simultaneous actions of photocharging and galvanostatic discharging. The battery in the dark had a capacity of 566 mAhg^–1^ at 0.2 V, while the PHBAT under illumination for 24 h had a capacity exceeding 1000 mAhg^–1^. Since the rate of photostorage is roughly equal to the discharge rate, the effective capacity could be increased arbitrarily by the illumination time. Once the illumination turns off, the supply of photogenerated charges is cut and the battery discharges normally in the dark. A table for comparison with other reported works can be found in Table S2.

The mechanism for photocharging in PHBATs of this type is depicted in Figure 4 and is similar to that described in previous works.^8,14^ In the diagram (Figure 4a), photogenerated electrons in Cs_2_NaBiI_6_ pass via PCBM to the CF current collector due to favorable band alignment when the PHBAT is irradiated with photons exceeding the band gap energy. The holes are kept in the valence band of the perovskite, where they can engage in one of two processes, depending on the device’s charged state. The first process is the repulsion by valence band holes toward the intercalated Li^+^, which can cause them to go back into the electrolyte via delithiation, leading to photocharging. Second, at lower voltages (after discharge) metallic bismuth can undergo photo-oxidation to Bi^3+^.^36^ After the liberation of the Li^+^ ion from the perovskite, the device then regains its charge to generate power. Transient absorption (TA) spectroscopy measurements (Figure S8) confirm some of the dynamics of the photogenerated charge carriers. These show an increasing lifetime of the Cs_2_NaBiI_6_ ground state bleach, due to charge separation between the Cs_2_NaBiI_6_ and PCBM, while PCBM and the conductive polymer alone show no response to the same excitation, on that time scale. Recent studies indicate two paths for the travel of photogenerated electrons.^8,9^ According to the conduction band energy diagram, it can either migrate towards the electrode and recombine with the hole, or it can travel through the external circuit to the other (counter) electrode and reduce the Li^+^ ions to Li metal, thus maintaining charge neutrality.^8^ There is another possibility, where the photogenerated electrons react with ethylene carbonate/dimethyl carbonate solvent present in the electrolyte, which leads to the formation of a reactive oxygen species (ROS), resulting in the production of a solid electrolyte interphase (SEI) on Li metal.^9^ The schematic explaining this phenomenon is shown in Figure 4c. Due to large energy differences in the photons collected and the lithium reduction potential, an external circuit will be needed eventually to reduce Li^+^ to Li^0^ and reconstitute the metal electrode. However, due to the large amount of Li metal initially available in the device, photocharging of the battery via reconstitution of the charged perovskite electrode can be observed over many cycles.

**Figure 4:**
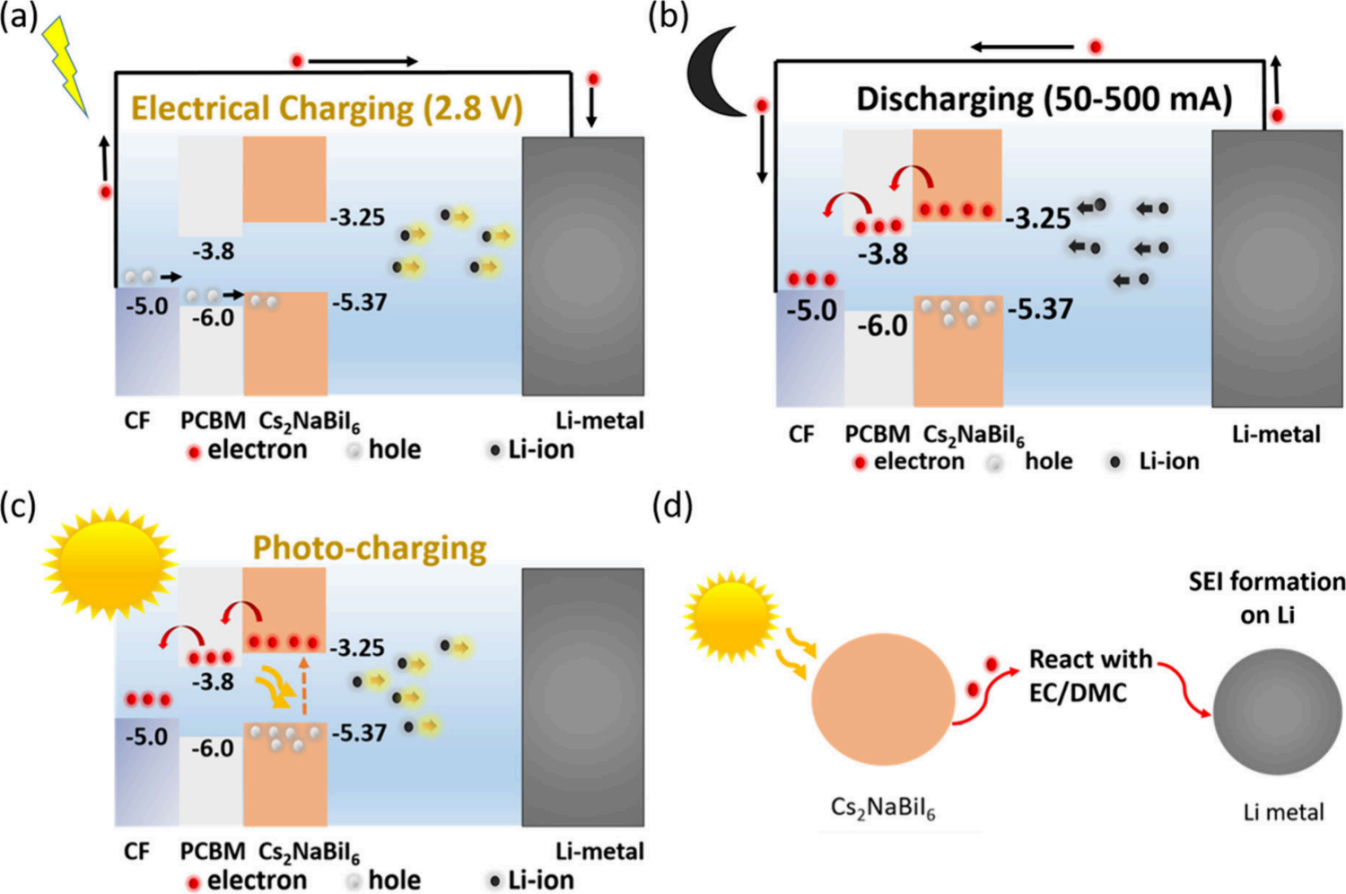
Photocharging mechanisms in the CF-PHBAT. (a) First charge mechanism showing the photocharge and (b) discharge mechanism, respectively, for the Cs_2_NaBiI_6_ photoanode, along with the energy level diagram. (c) Mechanism for photocharging and (d) second mechanism for electron reaction to cause SEI formation when the battery is in the open circuit state.

## 3 Conclusion

This study successfully showcases the first use of a lead-free double-perovskite halide, Cs_2_NaBiI_6_, as both an electrode and photoelectrode in lithium-ion photobatteries. The material exhibits remarkable electrochemical performance, achieving an initial specific capacity of 450 mAhg^–1^ and maintaining 150 mAhg^–1^ after 90 cycles, with a stable performance over 500 cycles. Ex situ XRD analysis indicates that the lithium storage mechanism involves the reduction of Bi^3+^ to metallic Bi^0^, followed by alloying events that produce LiBi and Li_3_Bi phases. The photo-rechargeable batteries (PHBATs) achieved an impressive maximum light conversion efficiency of 0.27% during the first discharge, representing the second highest efficiency reported for lithium-ion perovskite photobatteries under 1 sun illumination. The photocharging ability is experimentally validated using chronoamperometry studies, demonstrating increased current under light, while comparative discharge evaluations show significantly enhanced capacity under light as compared to that in the dark. The PHBAT efficiently powers a 1.5 V digital stopwatch for several minutes by photogenerated charging alone, demonstrating its practical utility. In comparison to our previous work on Cs_3_Bi_2_I_9_, the nonphoto Cs_2_NaBiI_6_ battery exhibits a significantly improved capacity (152 vs 47 mAhg^–1^ after 90 cycles), enhanced rate capability, and remarkable cycling stability, thereby establishing this double perovskite as a promising candidate for sustainable, high-performance photo-rechargeable energy storage systems.

## 4 Methods

### Materials

Bismuth iodide (BiI_3_ 99.99%), cesium iodide (CsI, 99.9% trace metals basis), sodium iodide (NaI), *N*-methyl-2-pyrrolidone (NMP, 99.5%), copper foil (Cu foil), poly­(vinylidene fluoride) (PVDF), and [6,6]-phenyl C61 butyric acid methyl ester (PCBM) were purchased from Sigma-Aldrich. Li metal foil, 1 M lithium hexafluorophosphate in ethylene carbonate, dimethyl carbonate with fluoroethylene carbonate (1 M LiPF_6_ in EC:DMC (1:1 in volume) with 5% FEC), and Whatman GF/A filter were purchased from DoDochem. Conductive carbon, Super P, was purchased from Timcal. Carbon felt (CF, Sigracet GDL 39 AA carbon graphite paper) was purchased from Fuel Cell Store. All the chemicals were used as received without further purification unless mentioned.

### Synthesis

The desired double-perovskite halide was synthesized using a one-step hydrothermal method.^37^ In 5 mL of 9 M hydroiodic acid (Sigma-Aldrich, 47 wt % in water), cesium iodide (CsI, Sigma-Aldrich, 99.999% trace metals basis), sodium iodide (NaI, Sigma-Aldrich, 99.999% trace metals basis), and bismuth iodide (BiI_3_, Sigma-Aldrich, 99.999 trace metals basis) were added in a stoichiometric ratio to obtain a 0.2 M Cs_2_NaBiI_6_ solution. After a short period of stirring, the solution was transferred to an autoclave. After 2 h of hydrothermal reaction at 120 °C, the product was manually cooled to room temperature at a constant rate over 10–12 h; deep red-colored crystals of Cs_2_NaBiI_6_ were obtained. To get the final product, the as-synthesized crystals were washed alternatively with minimal DI water, hexane, and IPA and centrifuged until the supernatant was colorless. Finally, the solution was dried in an oven at 40 °C to obtain the end product. Purified microcrystals were characterized via X-ray diffraction (XRD) by comparison with previous works and known standards (Figure S9).

### Electrode Fabrication on Cu Foil

70 mg amount of Cs_2_NaBiI_6_ powder obtained is hand-milled using a pestle and mortar along with 20 mg of conductive carbon and 10 mg of polyvinylidene fluoride (PVDF). It is then added to 500 μL of 1-methyl-2-pyrrolidinone (NMP) solvent and stirred overnight to obtain a thick slurry. The slurry is doctor-bladed uniformly onto copper foil and dried at 80 °C in an oven for 12 h to evaporate the solvent. The working electrodes are obtained by punching round discs of 17 mm diameter in the dried electrode foil. The real mass loading of Cs_2_NaBiI_6_ is from 1.1 to 1.3 mg/cm^2^.

### Electrode Fabrication on CF Foil

PCBM (20 mg) is dissolved in 1 mL of NMP by sonication for an hour. It is followed by dissolving 70 mg of the perovskite powder and stirring for 12 h at 1500 rpm. A 10 mg amount of PVDF is then added, and the solution is stirred for 1 h. We punch round discs of diameter 17 mm of CF and drop cast 40 μL of the solution, followed by drying it overnight at 80 °C.

### Fabrication of Photobatteries

The process is carried out inside an argon glovebox. We modified the top of the CR2450 coin cell by punching a 9 mm hole in the center to allow light to pass through. A transparent glass substrate is glued to it to allow for light illumination. The coin cell is assembled with the Cs_2_NaBiI_6_ electrode, Whatman glass-fiber filter paper (GF/D) soaked in 1 M LiPF_6_ in EC/DMC with 5% FEC electrolyte, and a lithium foil chip (0.45 mm) and sealed. Areal mass loading of Cs_2_NaBiI_6_ in the photoelectrode is from 1.1 to 1.5 mg/cm^2^.

The photobattery (PHBAT) consists of a layer-by-layer assembly: current-collector (CF)/Cs_2_NaBiI_6_ photoelectrode/separator dipped in electrolyte/Li metal as the cathode. A xenon lamp (Perfect PLS-SXE300) at 1 sun was used as the light source unless otherwise mentioned.

## Supplementary Material



## Notes

Device schematic, additional performance measurements, ex situ XRD characterization, SEM images, efficiency calculations and table of LCE values for subsequent discharge, TA measurements, reference spectra for XRD analysis, and table of related previous works (PDF)
