# Supplementary material for: Photo-rechargeable Li-Ion Batteries with Lead-Free Double-Perovskite Halide Cs2NaBiI6
Source: ACS Appl Mater Interfaces. 2025 Jul 28;17(31):44360–7. doi: 10.1021/acsami.5c06043 (PMC12332828; doi:10.1021/acsami.5c06043)
Supplement: Supplementary file 1 [file am5c06043_si_001.pdf]

Supporting Information

# Photo-rechargeable Li-ion batteries with Lead-free Double Perovskite Halide $Cs_2NaBiI_6$

*Neha Tewari, Davy Lam, Pui Kei Ko, Pai Geng, Herman H.Y. Sung, C.-H. Angus Li, Ian Duncan Williams and Jonathan E. Halpert**

*Department of Chemistry, Hong Kong University of Science and Technology, Clear Water Bay Road, Kowloon, Hong Kong SAR 999077.*

*Corresponding Author Email: jhalpert@ust.hk

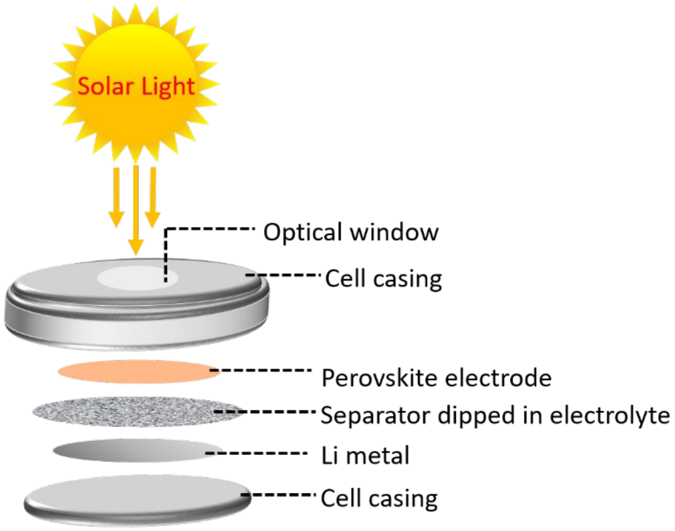


**Figure S1:** Schematic illustration of the PHBAT assembly. A schematic illustration of the electrochemical photo-coin cell used for measuring the electrochemical properties of the perovskite electrodes in a PHBAT set up.

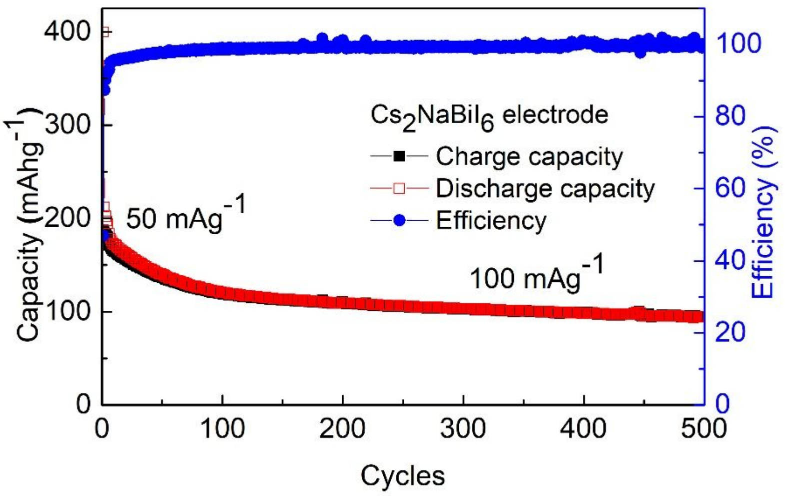


**Figure S2:** Long-term performance of $Cs_2NaBiI_6$ as anode for 500 cycles at 100 $mAg^{-1}$.

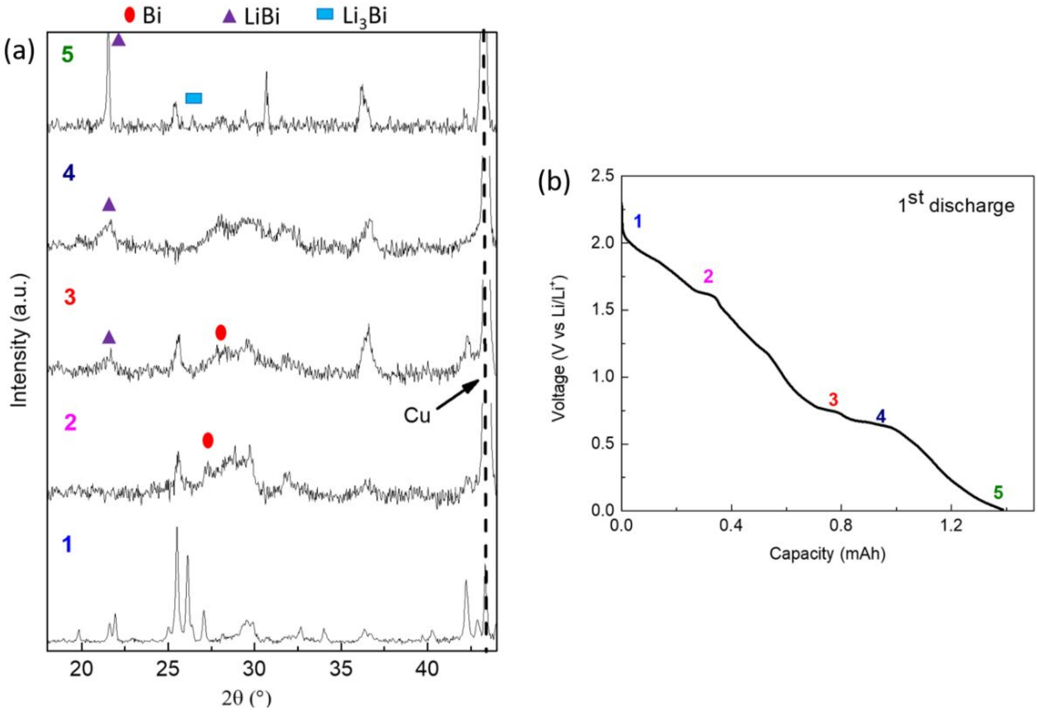


**Figure S3:** (a) Ex-situ XRD characterization at various stages of the first discharge cycle for $Cs_2NaBiI_6$ as anode in a coin cell with Li counter electrode, (b) discharge cycle showcasing the various points at which the XRD is analyzed.

Ex-situ X-ray diffraction (XRD), has shown to be effective in analyzing the lithiation process in a variety of electrode materials, and was conducted to investigate the mechanism of lithium storage. The cells were discharged until different potential points of the first discharge cycle at 100 $mAg^{-1}$ prior to being disassembled for analysis. After dissembling the cells, the electrodes were rinsed with DMC (dimethyl carbonate) to wash of the Li-based deposition on its surface and dried at 60°C before the analysis.

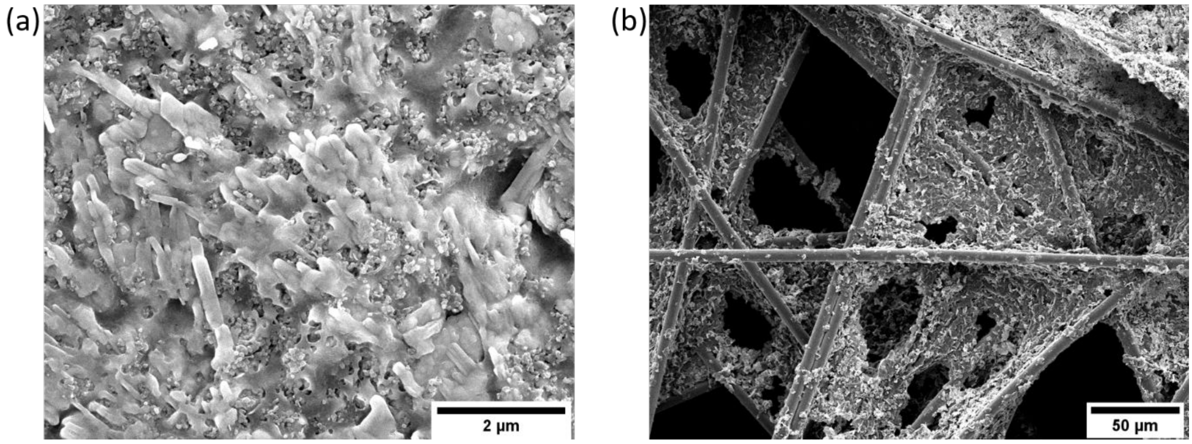


**Figure S4:** SEM image of $Cs_2NaBiI_6$ anode. (a) when doctor-bladed on copper foil, (b) when drop-casted onto the fibrous CF current collector.

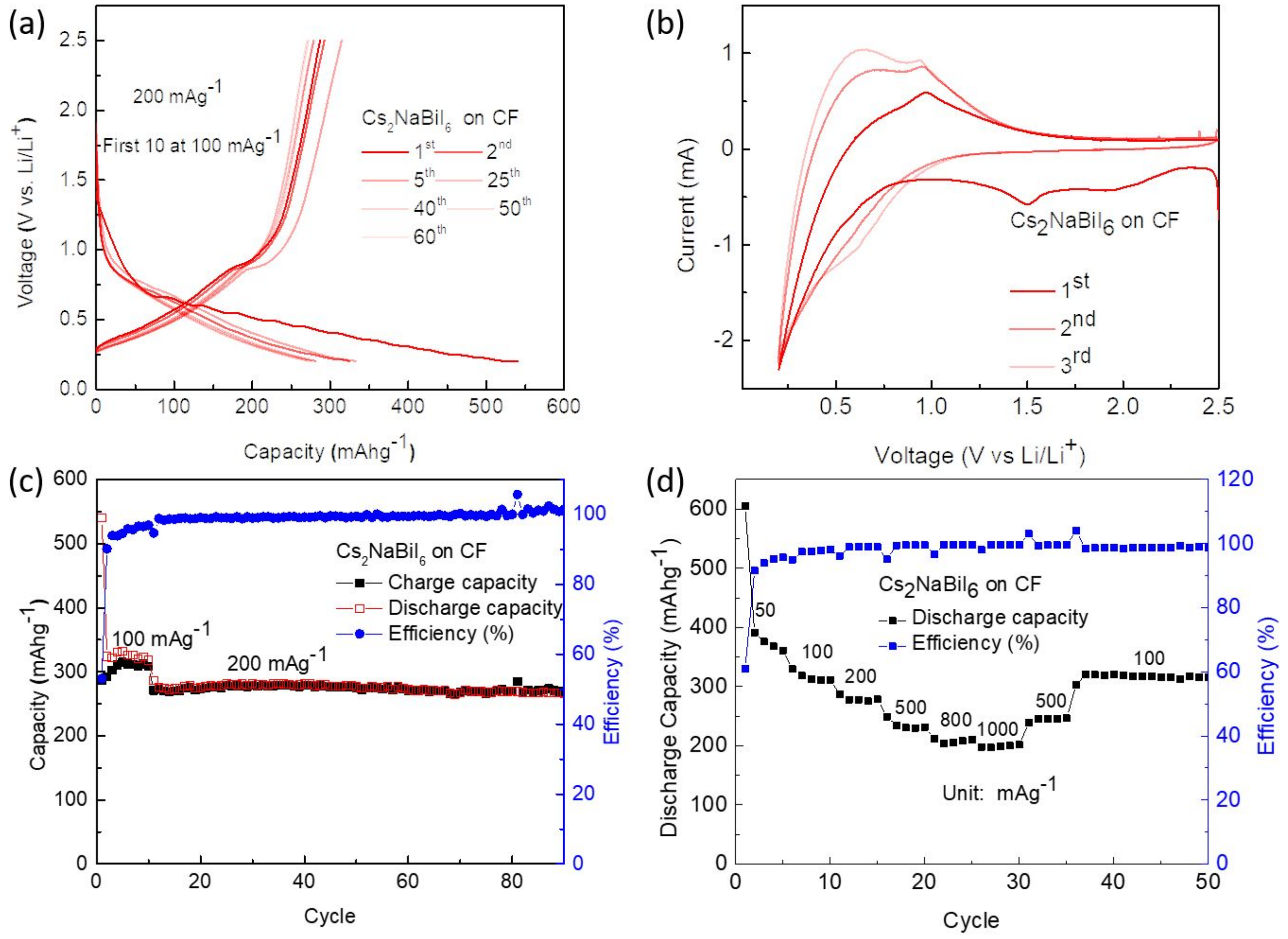


**Figure S5:** Electrochemical performance of $Cs_2NaBiI_6$ based lithium-ion (non-photo) batteries with a CF as current collector. (a) Galvanostatic charge-discharge curves in the voltage range of 2.5- 0.2 V vs $Li/Li^+$ at 200 $mAg^{-1}$ with the first 10 cycles at 100 $mAg^{-1}$, (b) Cyclic voltammetry scans of $Cs_2NaBiI_6$ vs. $Li/Li^+$ for the first 3 cycles at a voltage range of 2.5-0.2 V vs $Li/Li^+$ at a scan rate of 1 $mVs^{-1}$, (c) Long-term cycle stability vs $Li/Li^+$ at 100 $mAg^{-1}$ with the first 5 cycles at 50 $mAg^{-1}$, (d) Rate performance at varied current densities.

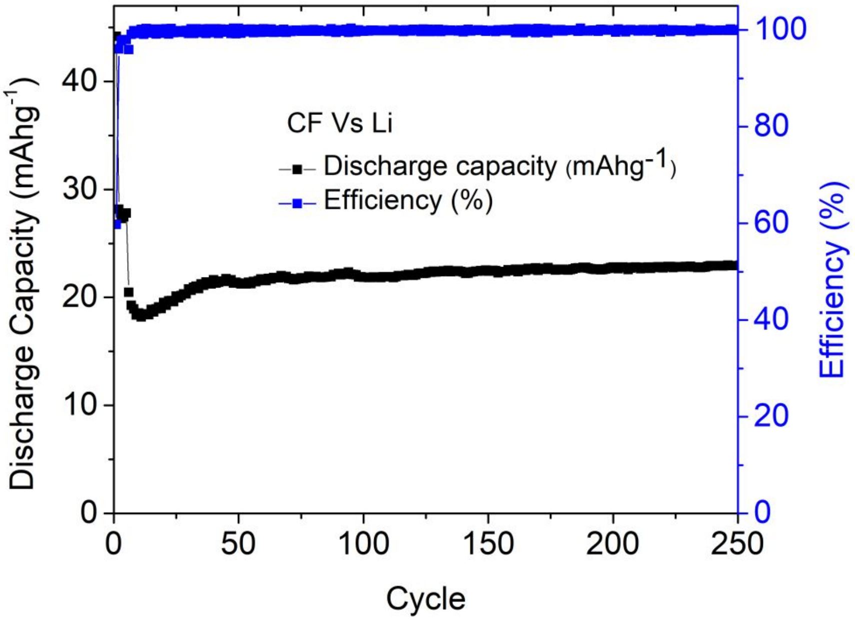


**Figure S6**: Electrochemical performance of CF vs Li for 250 cycles.

## Light conversion efficiency:

Light conversion efficiency is the efficiency of energy conversion and storage processes performed by PHBATs made from a photo-active material that can directly be charged by light without using solar cells. The calculation is shown below:

Light conversion efficiency data and calculations are included in Figure 3e and Table S1.

Efficiency Calculation shown:

The light conversion efficiency[1] is calculated using, Eq. S1: [1]

$$\eta = \frac{E_{output}}{E_{input}} = \frac{E_B B_1}{P_{in} T B_2} \qquad \text{(Equation S1)}$$

where $E_B$ = Areal energy density

$B_1$ = Surface area of the photo-battery which is 2.27 cm$^2$ for CF-PHBAT

$P_{in}$= Illuminated light density (100 mW/cm$^2$)

$T$ = Photo-charging time (h)

$B_2$ = Surface area of the illuminated area = 0.636 cm$^2$

The calculation for the 1$^{st}$ discharge/Photo-charge for CF-PHBAT:

Here, $E_B$ is obtained from the area under the areal capacity-voltage curves (Figure S7)

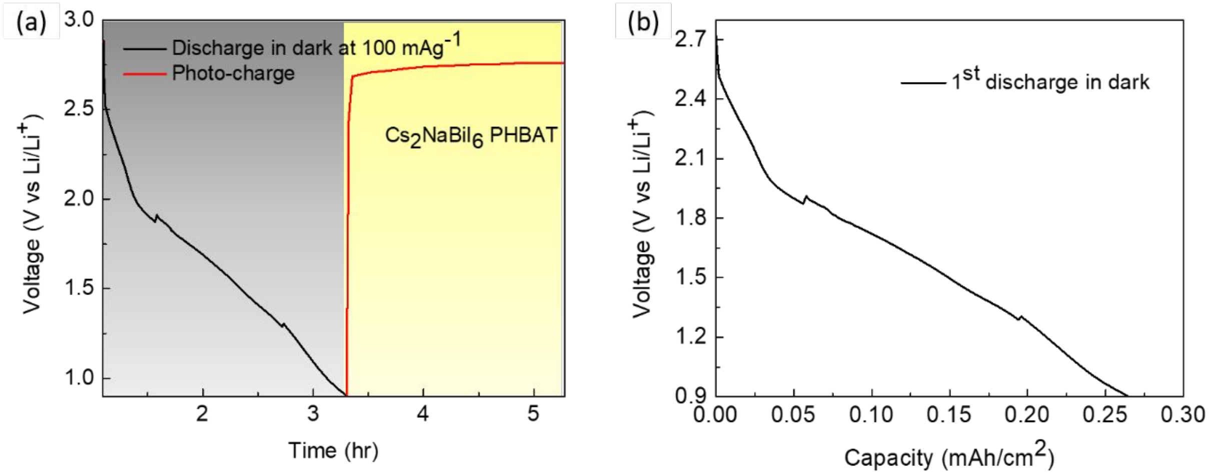


**Figure S7:** (a) The 1st discharge/photo-charge profile for $Cs_2NaBiI_6$ PHBAT using CF as the current collector, (b) the voltage-areal capacity discharge curve of the 1$^{st}$ discharge, integration of which finds the areal energy density used for the calculation of light-conversion efficiency

**Table S1:** LCE values obtained for $Cs_2NaBiI_6$ CF-PHBAT.

| CF-PHBAT | Areal Capacity (mAh/cm$^2$) | EB (mWh/cm$^2$) | Pin (mW/cm$^2$) | T (hr) | LCE (%) |
|---|---|---|---|---|---|
| 1$^{st}$ discharge/Photocharge | 0.26467 | 0.1805 | 100 | 2.431 | 0.265 |
| 2$^{nd}$ discharge/Photocharge | 0.06617 | 0.0272 | 100 | 1.1.04 | 0.088 |
| 3$^{rd}$ discharge/Photocharge | 0.0434 | 0.0142 | 100 | 1.135 | 0.045 |
| 4$^{th}$ discharge/Photocharge | 0.0365 | 0.0119 | 100 | 1.047 | 0.040 |
| 5$^{th}$ discharge/Photocharge | 0.03022 | 0.0102 | 100 | 0.9078 | 0.040 |

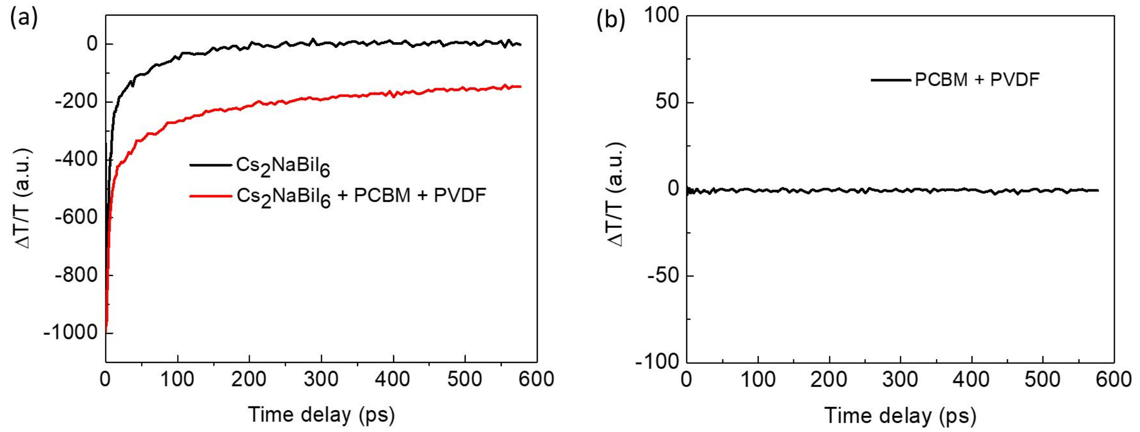


**Figure S8:** TA kinetics of the ground state bleach region compared to different combinations consisting of: a) pristine $Cs_2NaBiI_6$, the composite electrode ($Cs_2NaBiI_6$ + PCBM + PVDF) and, b) PCBM + PVDF.

**Figure S8a** shows the charge carrier dynamics on picosecond (ps) time intervals for the $Cs_2NaBiI_6$ and the composite sample, which consists of $Cs_2NaBiI_6$, PCBM, and PVDF. Adding PCBM in the composite sample extends the lifetime of the photo-excited charge carriers as PCBM can help in longer separation of photo-excited electrons and holes due to transfer of photo-generated electrons from $Cs_2NaBiI_6$ to PCBM (as shown in Figure 4a). The TA for the PCBM+PVDF composite in Figure S8b shows negligible TA signal confirming that PCBM by itself does not contribute to the photo-charging effect but facilitates the charge carrier transport from $Cs_2NaBiI_6$.

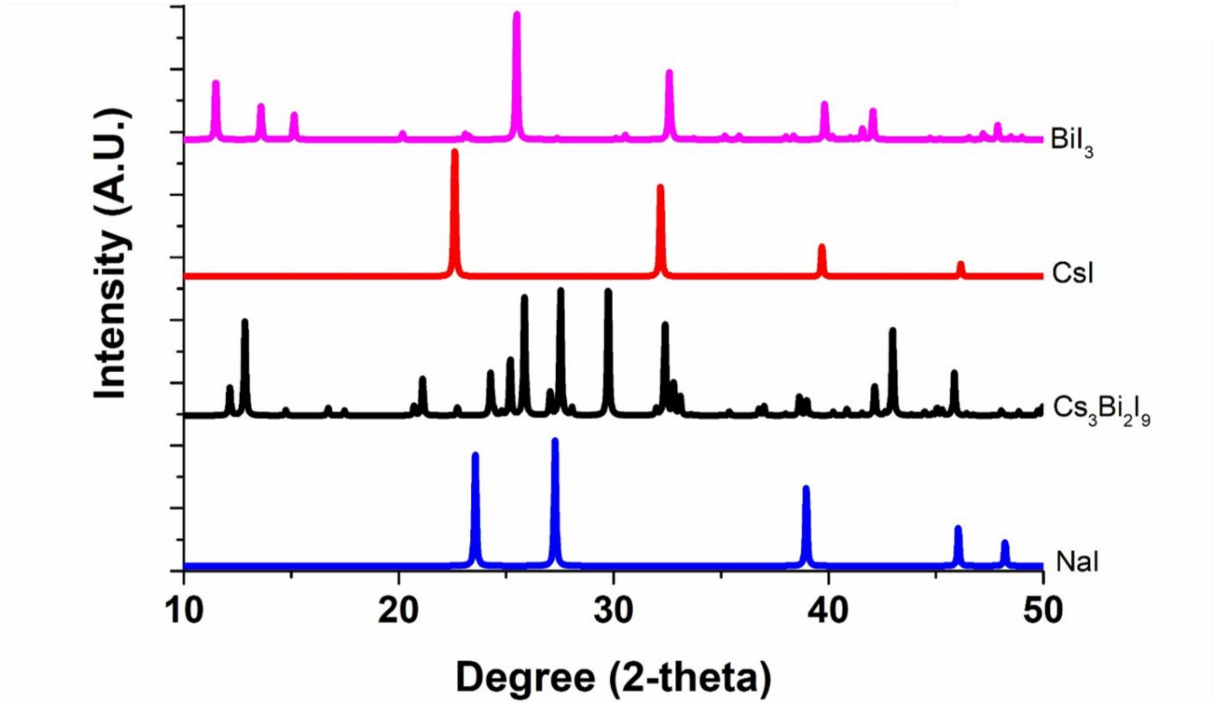


**Figure S9:** Standard references for $BiI_3$, CsI, $Cs_3Bi_2I_9$, and NaI for analysis of impurities and comparison.

Standard references replotted from open source (via "Materials Project"), respectively:

*[Xiao-Xiao Sun, Zhi-Ru Ren, and Dao-Guang Wang. Structural Transitions of BiI3 Under Pressure. Modern Physics Letters B, 26:1250217–1–1250217–8, 2012.]*

*[M. Blackman and I.H. Khan. The Polymorphism of Thallium and Other Halides at Low Temperatures. Proceedings of the Physical Society, London, 77:471–475, 1961.]*

*[A.V. Arakcheeva, M. Bonin, G. Chapuis, and A.I. Zaitsev. The Phases of $Cs_3Bi_2I_9$ Between RT and 190 k. Zeitschrift fuer Kristallographie (1979-2010), 214:279–283, 1999.]*

*[P. Cortona. Direct Determination of Self-consistent Total Energies and Charge Densities of Solids: a Study of the Cohesive Properties of the Alkali Halides. Physical Review, Serie 3. B - Condensed Matter (18,1978-), 46:2008–2014, 1992.]*

CNBI standards were obtained from the following sources (not reprinted here due to copyright):

*[Li et al. Physical Stata Solidi A, 2019, 216 (23), 1900567; Zheng et al. Journal of Alloys & Compounds 2022, 909, 164613; Zhang et al. Sustainable Energy Fuels, 2018,2, 2419-2428; Cheng et al. New J. Chem., 2017,41, 9598-9601].*

**Table S2**: Comparison table with other reported work.

| Photoelectrode | Battery type | Light source | LCE (%) | Ref. |
|---|---|---|---|---|
| $Cs_2NaBiI_6$ | Li-ion | 1 sun | 0.27 | This work |
| $Cs_3Bi_2I_9$ | Li-ion | 1 sun | 0.43 | [2] |
| $(C_6H_9C_2H_4NH_3)_2PbI_4$ | Li-ion | 1 sun | 0.034 | [3] |
| $LiFePO_4$-Ru dye | Li-ion | 1 sun | 0.06 | [4] |
| $V_2O_5$/P3HT | Li-ion | 1 sun<br>455 nm | 0.22<br>2.6 | [5] |
| $Fe_2O_3$ | Li-ion | 470 nm | 1.98 | [6] |
| $MoS_2/MoO_x$ | Li-ion | 1 sun | 0.05 | [7] |
| $LiV_2O_5$ | Li-ion | 33 $mW/cm^2$ | 9 | [8] |
| $MoS_2$/ZnO | Zn-ion | 1 sun<br>455 nm | 0.2<br>1.8 | [9] |
| $VO_2$/ZnO | Zn-ion | 1 sun<br>455 nm | 0.2<br>0.51 | [10] |

**Supporting Information References:**


1. Liu, R.; Wang, J.; Sun, T.; Wang, M.; Wu, C.; Zou, H.; Song, T.; Zhang, X.; Lee, S.-T.; Wang, Z. L. Silicon Nanowire/Polymer Hybrid Solar Cell-supercapacitor: a Self-charging Power Unit with a Total Efficiency of 10.5%. *Nano Lett.* **2017,** 17, 7, 4240-4247.
2. Tewari, N.; Shivarudraiah, S. B.; Halpert, J. E. Photorechargeable Lead-free Perovskite Lithium-ion Batteries Using Hexagonal $Cs_3Bi_2I_9$ Nanosheets. *Nano Lett.*, **2021,** 21, 13, 5578-5585.
3. Ahmad, S.; George, C.; Beesley, D. J.; Baumberg, J. J.; De Volder, M. Photo-rechargeable Organo-halide Perovskite Batteries. *Nano Lett.* **2018,** 18, 3, 1856-1862.
4. Paolella, A.; Faure, C.; Bertoni, G.; et al. Light-assisted Delithiation of Lithium Iron Phosphate Nanocrystals Towards Photo-rechargeable Lithium Ion Batteries. *Nature*, **2017,** 8, 1, 14643.
5. Boruah, B. D.; Mathieson, A.; Wen, B.; Feldmann, S.; Dose, W. M.; De Volder, M. Photo-rechargeable Zinc-ion Batteries. *Energy Environ. Sci.* **2020,** 13, 8, 2414-2421.
6. Chamola, S.; Ahmad, S. High Performance Photorechargeable Li-Ion Batteries Based on Nanoporous $Fe_2O_3$ Photocathodes. *Adv. Sustainable Syst.*, **2023,** 7, 6, 2300043.
7. Kumar, A.; Thakur, P.; Sharma, R.; Puthirath, A. B.; Ajayan, P. M.; Narayanan, T. N. Photo Rechargeable Li-Ion Batteries Using Nanorod Heterostructure Electrodes. *Small* **2021,** 17, 51, 2105029.
8. Wang, J.; Wang, Y.; Zhu, C.; Liu, B. Photoinduced Rechargeable Lithium-ion Battery. *ACS Appl. Mater. Interfaces* **2022,** 14, 3, 4071-4078.
9. Boruah, B. D.; Wen, B.; De Volder, M. Molybdenum Disulfide–zinc Oxide Photocathodes for Photo-rechargeable Zinc-ion Batteries. *ACS Nano* **2021,** 15, 10, 16616-16624.
10. Boruah, B. D.; De Volder, M., Vanadium Dioxide–zinc Oxide Stacked Photocathodes for Photo-rechargeable Zinc-ion Batteries. *J. Mater. Chem. A* **2021,** 9, 40, 23199-23205.
